# Interstitielle Lungenerkrankungen bei Kollagenosen: Ein musterbasierter Ansatz zur Diagnostik

**DOI:** 10.1007/s00117-025-01492-4

**Published:** 2025-08-22

**Authors:** Rebecca Mura, Daria Kifjak, Kastriot Kastrati, Nino Bogveradze, Lucian Beer, Helmut Prosch

**Affiliations:** 1 Univ. Klinik für Radiologie und Nuklearmedizin, Medizinische Universität Wien, Währinger Gürtel 18–20, 1090 Wien, Österreich; 2 Abteilung für Rheumatologie, Universitätsklinik für Innere Medizin III, Medizinische Universität Wien, 1090 Wien, Österreich

**Keywords:** Bindegewebserkrankungen, Gewöhnliche interstitielle Pneumonie, Nichtspezifische interstitielle Pneumonie, Autoimmunerkrankungen, Hochauflösende Computertomographie, Connective tissue diseases, Common interstitial pneumonia, Non-specific interstitial pneumonia, Autoimmune diseases, High-resolution computed tomography

## Abstract

Bindegewebserkrankungen („connective tissue diseases“, CTD) umfassen eine heterogene Gruppe systemischer, immunvermittelter Erkrankungen, die das Bindegewebe im gesamten Körper betreffen. Eine pulmonale Beteiligung ist eine häufige und klinisch bedeutsame Manifestation von CTD, wobei die interstitielle Lungenerkrankung (ILD) einen wesentlichen Beitrag zur Morbidität und Mortalität leistet. Daher ist die frühzeitige Erkennung von CTD-ILD von entscheidender Bedeutung, und ein multidisziplinärer Ansatz ist sowohl für die Diagnose als auch für das Patientenmanagement von größter Wichtigkeit. In diesem Zusammenhang spielt die hochauflösende Computertomographie (HRCT) eine zentrale Rolle – nicht nur bei der Identifizierung charakteristischer ILD-Muster, sondern auch bei der Verlaufskontrolle und der therapeutischen Entscheidungsfindung. Obwohl das häufigste bildgebende Muster bei CTD-ILD die nichtspezifische interstitielle Pneumonie (NSIP) ist, zeigt sich das Spektrum pulmonaler Manifestationen komplex und heterogen. Weitere Muster wie die gewöhnliche interstitielle Pneumonie (UIP), die organisierende Pneumonie (OP), die lymphozytische interstitielle Pneumonie (LIP) und die diffuse alveoläre Schädigung (DAD) können ebenfalls auftreten und zeigen jeweils spezifische Assoziationen mit bestimmten CTD. Darüber hinaus sind Überlappungen und Übergänge zwischen den Mustern nicht selten, was die Diagnosestellung zusätzlich erschwert. Diese Übersichtsarbeit zielt darauf ab, einen umfassenden Überblick über die wichtigsten pulmonalen Manifestationen von CTD, insbesondere der ILD, zu geben – mit Schwerpunkt auf der HRCT-basierten Mustererkennung, um die diagnostische Sicherheit und das Musterverständnis der Radiologinnen zu verbessern.

## Abstract (en)

Connective tissue diseases (CTD) encompass a heterogeneous group of systemic immune-mediated disorders affecting connective tissue throughout the body. Pulmonary involvement is a common and clinically significant manifestation of CTD, with interstitial lung disease (ILD) representing a major contributor to both morbidity and mortality. Therefore, early detection of CTD-ILD is critical, and a multidisciplinary approach is paramount for both diagnosis and patient management. In this context, high-resolution computed tomography (HRCT) plays a pivotal role not only in recognizing characteristic ILD patterns but also in monitoring disease progression and guiding further management. Although the most frequent imaging pattern in CTD-ILD is nonspecific interstitial pneumonia (NSIP), the spectrum of pulmonary manifestations is complex and heterogeneous. Other patterns such as usual interstitial pneumonia (UIP), organizing pneumonia (OP), lymphocytic interstitial pneumonia (LIP), and diffuse alveolar damage (DAD) can also occur, showing distinct associations with specific CTD. Moreover, overlap and transition between patterns are not uncommon, further complicating the diagnostic process. This review aims to provide a comprehensive overview of the most relevant pulmonary manifestations of CTD, particularly ILD, with a focus on HRCT-based pattern recognition in order to enhance radiologist’s familiarity with their spectrum of imaging appearances.

## Hintergrund

Der Begriff der Kollagenosen („connective tissue diseases“, CTD) umfasst eine heterogene Gruppe systemischer, immunmediierter Erkrankungen, die typischerweise durch chronische Entzündung, Gewebeschädigung und abnorme Reparaturprozesse gekennzeichnet sind, welche zur Ablagerung von fibrotischem Gewebe und schließlich zum Funktionsverlust der betroffenen Organe führen können [1–3]. Dabei handelt es sich um Autoimmunerkrankungen, deren Pathogenese sowohl durch genetische als auch durch Umweltfaktoren beeinflusst wird [1]. Zu den häufigsten CTD zählen systemische Sklerose (SSc), idiopathische inflammatorische Myopathien (IIM), systemischer Lupus erythematodes (SLE), das Sjögren-Syndrom (SjD) und gemischte Bindegewebserkrankungen (MCTD; [1–3]; Tab. 1). Obwohl die rheumatoide Arthritis (RA) nicht im engeren Sinne zu den Kollagenosen zählt, wird sie üblicherweise den CTD zugeordnet.

**Tab. 1:** Wichtige serologische Marker und Befunde der hochauflösenden Computertomographie (HRCT) der Lunge bei den häufigsten Kollagenosen

CTD	Marker	HRCT-Befunde der Lunge
*RA*	Anti-CCP, RF	UIP-, NSIP-Muster Atemwegserkrankung (obliterative Bronchiolitis) Rheumatoide Knötchen
*SSc*	Anti-Scl70, Anti-RNA Polymerase, ANA	NSIP-, UIP-Muster; selten, OP, LIP Pulmonale Hypertension
*IIM*	MSAs: Anti-tRNA synthetase (Jo‑1, PL7, PL12, EJ, OJ, KS, ZO, HA) MDA5; MAAs (Ro-52); KU; PM/SCL	NSIP/OP-, UIP-Muster; DAD Hypoventilation infolge einer Beteiligung der Atemmuskulatur Aspirationspneumonie
*SLE*	Anti-dsDNA, Anti-Smith, Lupus anticoagulant, ANA	NSIP-Muster Akute Lupus-Pneumonitis, DAH, „shrinking lung syndrome“ Opportunistische pulmonale Infektionen Pulmonale Thromboembolie, pulmonale Hypertension
*SjD*	Anti-SS‑A (Ro60-Ro52) Anti-SS-B/La, ANA	NSIP-, LIP-Muster; OP und UIP Atemwegsbeteiligung (follikuläre Bronchiolitis)
*MCTD*	Anti-Ro52, Anti-U1-RNP	NSIP-, OP-, UIP-Muster; DAD gelegentlich beobachtet

*CTD* „connective tissue disease“ (Kollagenose), *RA* rheumatoide Arthritis, *CCP* Antikörper gegen zyklisch citrullinierte Peptide, *RF* Rheumafaktor, *SSc* systemische Sklerose, *IIM* idiopathische entzündliche Myopathie, *MSA* Myositis-spezifische Autoantikörper, *MAA* Myositis-assoziierte Antikörper, *SLE* systemischer Lupus erythematodes, *ANA* antinukleäre Antikörper, *SjD* Sjögren disease (Sjögren-Syndrom), *MCTD* „mixed connective tissue disease“ (Mischkollagenose), *UIP* „usual interstitial pneumonia“ (gewöhnliche interstitielle Pneumonie), *NSIP* „non-specific interstitial pneumonia“ (nichtspezifische interstitielle Pneumonie), *OP* „organizing pneumonia“ (organisierende Pneumonie), *LIP* „lymphocytic interstitial pneumonia“ (lymphozytäre interstitielle Pneumonie), *DAD* „diffuse alveolar damage“ (diffuser alveolärer Schaden)

Alle thorakalen Kompartimente – Lungenparenchym, Pleura, Atemwege, Gefäße und Thoraxwand – können in unterschiedlichem Ausmaß und in verschiedenen Kombinationen im Rahmen einer CTD betroffen sein, wobei interstitielle Lungenerkrankungen (ILD) zu den häufigsten und klinisch relevantesten Manifestationen zählen [1–3].

Diese Übersicht stellt die wichtigsten pulmonalen Manifestationen der CTD, insbesondere die ILD, dar und zielt darauf ab, Radiologinnen und Radiologen mit den häufigsten radiologischen Mustern dieser Erkrankungen vertraut zu machen.

## Diagnostik

Im Allgemeinen kann die ILD nach der Diagnose einer CTD, gleichzeitig mit ihr oder sogar Jahre vor der klinischen Diagnose auftreten [4]. Daher gestaltet sich die genaue Erfassung der Epidemiologie der parenchymalen Lungenbeteiligung schwierig. Die Angaben zur Inzidenz und Prävalenz variieren nicht nur je nach zugrunde liegender CTD, sondern auch in Abhängigkeit von den angewendeten Diagnosekriterien und verwendeten Untersuchungsmethoden [4]. Die hohe Prävalenz von Überlappungssyndromen und undifferenzierten CTD stellt eine zusätzliche diagnostische Herausforderung dar [3].

Für die RA wurden beispielsweise in zwei großen populationsbasierten Studien Inzidenzraten der RA-ILD zwischen etwa 2 und 7 % angegeben [5, 6], wobei asymptomatische oder subklinische ILD in diesen Schätzungen nicht berücksichtigt wurden [4]. Bei der SSc zeigt sich die pulmonale Beteiligung häufiger als bei der RA. Eine systematische Übersichtsarbeit von Bergamasco et al. berichtete eine jährliche Inzidenz und Prävalenz der SSc-ILD in Europa von 0,7–4,2 bzw. 0,1–0,4 pro 100.000 [7].

Die Diagnose einer CTD-ILD erfordert eine umfassende Beurteilung der klinischen Merkmale, die durch eine multidisziplinäre Fallkonferenz mit Radiologinnen, Pneumologinnen, Pathologinnen und Rheumatologinnen optimiert werden kann [8, 9]. Während der Nutzen von multidisziplinären Fallkonferenzen bei ILD allgemein anerkannt ist [8, 10], gibt es bislang nur wenige Studien, die sich spezifisch mit multidisziplinären Fallkonferenzen im Zusammenhang von CTD-ILD befassen [10, 11]. Dennoch kann die multidisziplinäre Fallkonferenz dazu beitragen, zuvor nicht erkannte CTD-ILD neu zu klassifizieren, was erhebliche Auswirkungen auf das Management und die Prognose haben kann [10, 11]. Zudem ermöglichen die multidisziplinären Fallkonferenzen die Festlegung einer klinischen Verdachtsdiagnose, anhand derer Patientinnen engmaschig überwacht und die Diagnose im Zeitverlauf überprüft werden können.

Klinische Manifestationen der CTD-ILD sind heterogen und reichen von subklinischen Verläufen (radiologisch nachweisbar, aber symptomfrei) über chronisch-progrediente bis hin zu rasch verlaufenden, lebensbedrohlichen Zuständen [12]. Die häufigste Symptomkonstellation besteht aus Allgemeinsymptomen und unspezifischen respiratorischen Beschwerden wie progredienter Belastungsdyspnoe, trockenem Husten und Thoraxschmerzen. In fortgeschrittenen Stadien können Hypoxie, Zyanose, Ödeme und Zeichen einer pulmonalen Hypertonie (PH) auftreten [13]. Auskultatorisch finden sich häufig basale Knisterrasselgeräusche (*Klettverschluss-Phänomen*), die typischerweise mit fibrotischen ILD-Formen korrelieren [13].

Ein wesentlicher Bestandteil der diagnostischen Abklärung bei Verdacht auf eine CTD ist der Nachweis von Autoantikörpern. Innerhalb des breiten Spektrums an serologischen Biomarkern wurden mehrere CTD-assoziierte Autoantikörper mit einem erhöhten Risiko für ILD in Verbindung gebracht [14, 15]. Bei Patientinnen mit SSc besteht eine starke Assoziation zwischen Anti-Scl70-Antikörpern und der Entwicklung einer ILD – vergleichbar mit der Bedeutung von Anti-CCP-Antikörpern bei der RA [15]. Bei Patientinnen mit IIM sind Antisynthetase-Antikörper mit einem hohen ILD-Risiko assoziiert, mit einer Prävalenz von bis zu 90 % [15, 16]. Anti-Ro52-Antikörper andererseits stehen in starker Verbindung zur MCTD-ILD [16]. Lungenfunktionsprüfungen (LFUs) sind ein hilfreiches Mittel zur Abschätzung der Schwere der Erkrankung und zur Verlaufskontrolle der CTD-ILD, insbesondere im Fall von RA und SSc [17]. Bei fibrotischen Formen der ILD zeigt sich typischerweise eine Restriktion [18].

Die Bildgebung spielt eine zentrale Rolle in der Diagnostik der CTD-ILD. Die konventionelle Röntgenaufnahme des Thorax ist aufgrund der geringen Kosten, der geringen Strahlenexposition und breiten Verfügbarkeit die Erstuntersuchung. Aufgrund der limitierten Sensitivität und Spezifität kann eine unauffällige Röntgenaufnahme ILD jedoch nicht zuverlässig ausschließen [19]. Daher ist die Durchführung einer hochauflösenden Computertomographie (HRCT) essenziell, um eine pulmonale Beteiligung festzustellen und das radiologische Muster einzuordnen [4].

Eine Lungenbiopsie (z. B. transbronchiale Kryobiopsie) ist zur Diagnosesicherung einer CTD-ILD meist nicht erforderlich, da die HRCT in der Regel ausreicht, um das Vorliegen und das Muster der Erkrankung zu identifizieren. Eine Biopsie kann jedoch indiziert sein, um andere Ursachen für pulmonale Verdichtungen oder Rundherde auszuschließen [20]. Das häufigste histopathologische Muster bei CTD-ILD entspricht – wie in der HRCT – der NSIP, mit Ausnahme der RA, bei welcher häufiger das UIP-Muster vorliegt [4]. Eine Bronchoskopie mit bronchoalveolärer Lavage (BAL) kann bei der Differenzialdiagnose überlappender Erkrankungen (z. B. alveoläre Hämorrhagie, eosinophile Infiltration oder Hypersensitivitätspneumonitis) hilfreich sein [20].

## Rolle der diagnostischen Bildgebung

### Thoraxröntgen

Die konventionelle Röntgenaufnahme des Thorax stellt bei Verdacht auf eine ILD häufig die erste bildgebende Untersuchung dar, da sie weit verbreitet, kostengünstig und mit einer geringen Strahlenexposition verbunden ist. Der diagnostische Wert ist jedoch stark eingeschränkt. Frühe interstitielle Veränderungen sind häufig zu subtil, um im Thoraxröntgen erkannt zu werden [19, 21]. Selbst bei klinischem Verdacht kann ein unauffälliges Thoraxröntgen eine ILD daher nicht zuverlässig ausschließen [19, 21]. In akuten Situationen (z. B. Dyspnoe) kann die Röntgenaufnahme jedoch hilfreich sein, um Differenzialdiagnosen wie z. B. eine Pneumonie oder eine Herzinsuffizienz zu stellen bzw. auszuschließen [21].

### Computertomographie

Die HRCT ist die wichtigste bildgebende Methode zur Diagnostik der ILD – nicht nur zum Nachweis der Erkrankung, sondern auch zur Identifikation radiologischer Muster, zur Abschätzung des Schweregrads und zur Verlaufskontrolle [3]. Der Untersuchungsstandard umfasst eine native, volumetrische Dünnschichtaufnahme des Thorax (1–1,5 mm Schichtdicke, um Partialvolumeneffekte zu minimieren), die in einer Atemanhaltephase gewonnen und mit einem hochauflösendem Rekonstruktionsalgorithmus berechnet wird [22]. Zusätzlich kann eine Exspirationsaufnahme durchgeführt werden, um Hinweise auf eine Mitbeteiligung der kleinen Atemwege zu erhalten. Da eine lageabhängige dorsale Atelektase frühe fibrotische Veränderungen imitieren kann, empfiehlt sich in bestimmten Fällen auch eine Akquisition in Bauchlage [23]. Multiplanare Rekonstruktionen (MPR) sind nützlich zur Beurteilung des Lungenvolumens und der Verteilung der Parenchympathologie in kraniokaudaler Richtung. Minimum-Intensitäts-Projektionen (MinIP) eignen sich zur Differenzierung von Honeycombing gegenüber ähnlichen Veränderungen wie Traktionsbronchiektasen [23].

Während diese Technik weiterhin den Standard in der ILD-Diagnostik darstellt, richtet sich zunehmendes Interesse auf Strategien zur Dosisreduktion. Obwohl sie zur Detektion subtiler ILD-Veränderungen nicht empfohlen wird, findet die Low-Dose-CT (LDCT) zunehmend Anwendung im klinischen Alltag [24]. Darüber hinaus ermöglicht die Einführung neuer Technologien wie der photonenzählenden Computertomographie (PCD-CT) eine Bildgebung mit ultrahoher Auflösung (bis 0,2 mm Schichtdicke) bei gleichzeitig niedriger Strahlendosis, was die Darstellung feiner Veränderungen sowie anatomischer Details deutlich verbessert [25, 26].

### Muster der CTD-ILD

Die HRCT hat die Diagnostik und das Management der ILD, insbesondere der fibrotischen Formen, grundlegend verändert. Es ist bekannt, dass die wichtigsten CT-Zeichen einer Fibrose aus retikulären Verdichtungen, Traktionsbronchiektasen und -bronchiolektasen, Honeycombing sowie Volumenverlust bestehen [27]. Ein weiteres häufiges HRCT-Merkmal bei ILD sind milchglasartige Verdichtungen („ground-glass opacities“, GGO), die typischerweise mit Retikulationen oder Traktionsbronchiektasen kombiniert auftreten und sowohl aktive Entzündungen als auch feinfibröse Veränderungen widerspiegeln können [28]. Die Kombination und Verteilung dieser Zeichen – axial wie auch kraniokaudal – definiert die jeweiligen HRCT-Muster der ILD.

Die bei CTD-ILD beobachteten radiologischen Muster ähneln jenen der idiopathischen interstitiellen Pneumonien (IIP; [12]). Allerdings werden bei CTD-ILD häufiger GGO sowie extraparenchymale Befunde (z. B. ösophageale Dilatation, Pleuraergüsse) beobachtet als bei IIP [29]. Das am häufigsten vorkommende Muster bei CTD ist die nichtspezifische interstitielle Pneumonie („nonspecific interstitial pneumonia“*,* NSIP), gefolgt von der gewöhnlichen interstitiellen Pneumonie *(„*usual interstitial pneumonia“, UIP), der organisierenden Pneumonie („organizing pneumonia“, OP) sowie – seltener – der lymphozytischen interstitiellen Pneumonie („lymphocytic interstitial pneumonia“, LIP) oder der diffusen alveolären Schädigung („diffuse, alveolar damage“, DAD) ([1, 4, 30, 31]; Tab. 2).

**Tab. 2:** Häufigkeit von Mustern in der hochauflösenden Computertomographie (HRCT) bei verschiedenen Kollagenosen. (Adaptiert nach Lynch, 2009 [30])

	RA	SSc	IIM	SLE	SjD	MCTD
UIP-Muster	+++	++	++	+	+	+
NSIP-Muster	++	+++	+++	+++	+++	+++
OP-Muster	+	+	+++	+	++	++
LIP-Muster	+	–	–	+	+++	–
DAD	+	+	+	–	–	+

*RA* rheumatoide Arthritis, *SSc* systemische Sklerose, *IIM* idiopathische inflammatorische Myopathie, *SLE* systemischer Lupus erythematodes, *SjD* Sjögren disease: Sjögren-Syndrom, *MCTD* „mixed connective tissue disease“ (Mischkollagenose), *UIP* „usual interstitial pneumonia“ (gewöhnliche interstitielle Pneumonie), *NSIP* „nonspecific interstitial pneumonia“ (nichtspezifische interstitielle Pneumonie), *OP* „organizing pneumonia“ (organisierende Pneumonie), *LIP* „lymphocytic interstitial pneumonia“ (lymphozytäre interstitielle Pneumonie), *DAD* „diffuse alveolar damage“ (diffuser alveolärer Schaden)

+++ häufige Assoziation, + relativ seltene Assoziation, *–* keine Assoziation

#### Nichtspezifische interstitielle Pneumonie

**Abb. 1:**
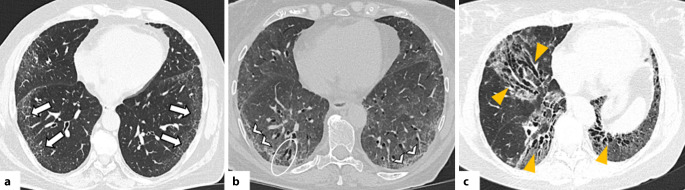
Axiale Aufnahmen der hochauflösenden Computertomographie (HRCT): Muster der nichtspezifischen interstitiellen Pneumonie (NSIP) bei Patienten mit systemischer Sklerose. **a** Milchglasverdichtungen, überwiegend in den peripheren Anteilen der Unterlappen, mit relativer subpleuraler Aussparung (*weiße Pfeile*). **b** Diffuse Milchglasverdichtungen und bilaterale periphere Retikulationen (*weiße Pfeilköpfe*), begleitet von Traktionsbronchiolektasen (*weißer Kreis*). **c** Fibrotischer NSIP-Subtyp: Milchglasverdichtungen in Mittel- und Unterlappen begleitet von zentralen Traktionsbronchiolektasen (*gelbe Pfeilköpfe*), die zentraler gelegen sind als beim Muster der gewöhnlichen interstitiellen Pneumonie (UIP). Zusätzlich zeigt sich in den Unterlappen ein ausgeprägter Volumenverlust

Das NSIP-Muster ist eine der häufigsten Formen der interstitiellen Pneumonie. Obwohl einige NSIP-Fälle idiopathisch sind, besteht eine enge Assoziation mit CTD, wobei die NSIP das häufigste histologische und radiologische Muster bei CTD-ILD darstellt [32]. Charakteristisch ist das gleichzeitige Vorliegen von Entzündung und ein variabler Grad an Fibrose, jedoch ohne die typische Heterogenität, wie sie bei UIP beobachtet wird [32]. Typische HRCT-Merkmale der NSIP sind symmetrische, basale milchglasartige Verdichtungen und feine retikuläre Zeichnungen, häufig begleitet von (zentralen) Traktionsbronchiektasen in der fibrotischen Subform [23, 33]. Eine überwiegende Beteiligung der Oberlappen ist bei NSIP untypisch und sollte differenzialdiagnostisch an andere Erkrankungen wie die Hypersensitivitätspneumonitis denken lassen [33]. Milchglasverdichtungen zeigen meist eine peribronchovaskuläre und zentral-periphere Verteilung, während die Traktionsbronchiektasen bei NSIP eher zentral als peripher lokalisiert sind, im Unterschied zur UIP [34]. Ein subpleurales Aussparen („subpleural sparing“), das als sehr typisch für NSIP gilt, findet sich jedoch nur in 21–30 % der Fälle ([23]; Abb. 1). Wenn fibrotische Merkmale wie Volumenverlust, Retikulationen oder Traktionsbronchiektasen vorliegen, spricht man von einer fibrotischen NSIP [23, 33]. Honeycombing ist bei der NSIP selten; falls es vorkommt, kann dies auf fokale UIP-Zonen hinweisen und als Zeichen einer Progression gewertet werden [33, 34].

Die NSIP kann gelegentlich mit der OP überlappen, insbesondere bei fibrotischen, peribronchial betonten Veränderungen mit subpleuraler Aussparung. Derzeit zeigen Studien keinen Überlebensunterschied zwischen idiopathischer und CTD-assoziierter NSIP [35].

#### Gewöhnliche interstitielle Pneumonie

Der Begriff UIP wurde ursprünglich zur Beschreibung des histopathologischen Korrelats der idiopathischen pulmonalen Fibrose (IPF) verwendet. Heute ist jedoch bekannt, dass das UIP-Muster nicht nur idiopathisch (im Rahmen der IPF), sondern auch sekundär im Rahmen von CTD (insbesondere RA), Pneumokoniosen (z. B. Asbestose), fibrotischer Hypersensitivitätspneumonitis und medikamententoxischen Reaktionen auftreten kann [36].

**Abb. 2:**
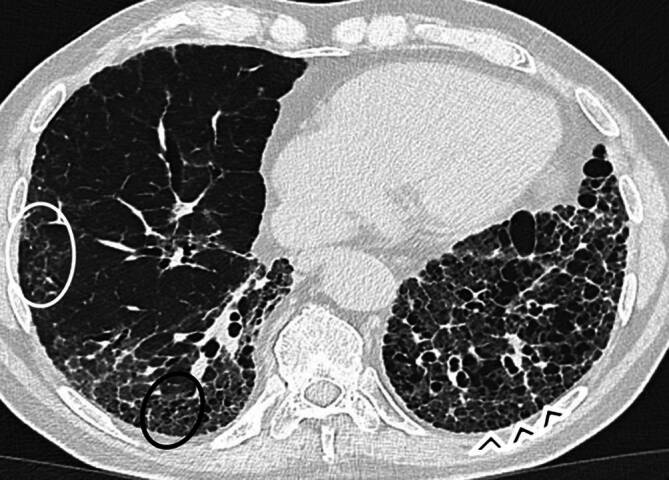
Axiale hochauflösende Computertomographie (HRCT) eines Patienten mit rheumatoider Arthritis (RA): Veränderungen entsprechend einem Muster der gewöhnlichen interstitiellen Pneumonie (UIP). Zu sehen sind Retikulationen (*weißer Kreis*), Bronchiolektasen (*schwarzer Kreis*) und Honigwabenbildung (*schwarze Pfeilköpfe*), überwiegend subpleural und basal lokalisiert. Die Milchglasverdichtungen, die mit Traktionsbronchiolektasen und Retikulationen auftreten, entsprechen in der Regel einer feinen Fibrose

Typische HRCT-Merkmale des UIP-Musters sind retikuläre Verdichtungen, Traktionsbronchiektasen und -bronchiolektasen, Volumenverlust und Honeycombing mit einer basalen und subpleuralen Verteilung (Abb. 2). Weitere Befunde können Milchglasverdichtungen sein – sie gelten zwar nicht als klassisches UIP-Zeichen, weisen in Kombination mit Traktionsbronchiektasen oder Retikulationen aber auf feinfibröse Veränderungen hin –, vergrößerte mediastinale Lymphknoten (> 10 mm) sowie lineare oder noduläre Verkalkungen in fibrotisch veränderten Arealen (im Sinne einer pulmonalen Ossifikation; [36, 37]).

Das positive prädiktive Potenzial der HRCT ist bei typischem UIP-Muster hoch – frühere Studien zeigten eine Übereinstimmung von bis zu 90–100 % zwischen radiologischer und histologischer UIP-Diagnose [38]. Da die Sensitivität der CT für UIP jedoch nur bei etwa 50 % liegt [38], unterteilt die Leitlinie der ATS/ERS/JRS/ALAT (American Thoracic/European Respiratory Society/Japanese Respiratory Society/Associatión Latinoamericana de Tórax) zur IPF-Diagnostik die HRCT-Muster in 4 Wahrscheinlichkeitskategorien ([22]; Tab. 3).

**Tab. 3:** Diagnostische CT-Kategorien der gewöhnlichen interstitiellen Pneumonie (UIP). (Adaptiert nach Raghu et al., 2018 [22])

	UIP	Wahrscheinliche UIP	Unbestimmte UIP	Alternative Diagnose
Hauptbefunde	Honeycombing, mit oder ohne periphere Traktionsbronchiektasen/-bronchiolektasen	Retikuläres Muster mit peripheren Traktionsbronchiektasen/Bronchiolektasen; kein Honeycombing	Subtile Retikulationen; milde GGO oder Architekturstörung	Hinweise auf eine andere Diagnose, z. B. Zysten, Mosaikperfusion, vorwiegende GGOs, zentrilobuläre Noduli, Konsolidierungen
Verteilung	Subpleural und basal betont	Subpleural und basal; oft heterogen	Subpleural und basal betont	Peribronchovaskulär, perilymphatisch, oberer/mittlerer Lungenabschnitt
Begleitbefunde	Milde GGO, retikuläres Muster, pulmonale Ossifikation	Milde GGO	CT-Merkmale und/oder Verteilung der Fibrose sprechen nicht für eine spezifische Ätiologie	Hinweise auf andere Diagnosen, z. B.:
				– Pleuraplaques (Asbestose)
				– Dilatierter Ösophagus (CTD)
				– Pleuraerguss/-verdickung (CTD/Medikamente)

*UIP* „usual interstitial pneumonia“ (gewöhnliche interstitielle Pneumonie), *GGO* „ground-glass opacity“ (Milchglasverdichtungen), *CTD* „mixed connective tissue disease“ (Mischkollagenose)

**Abb. 3:**
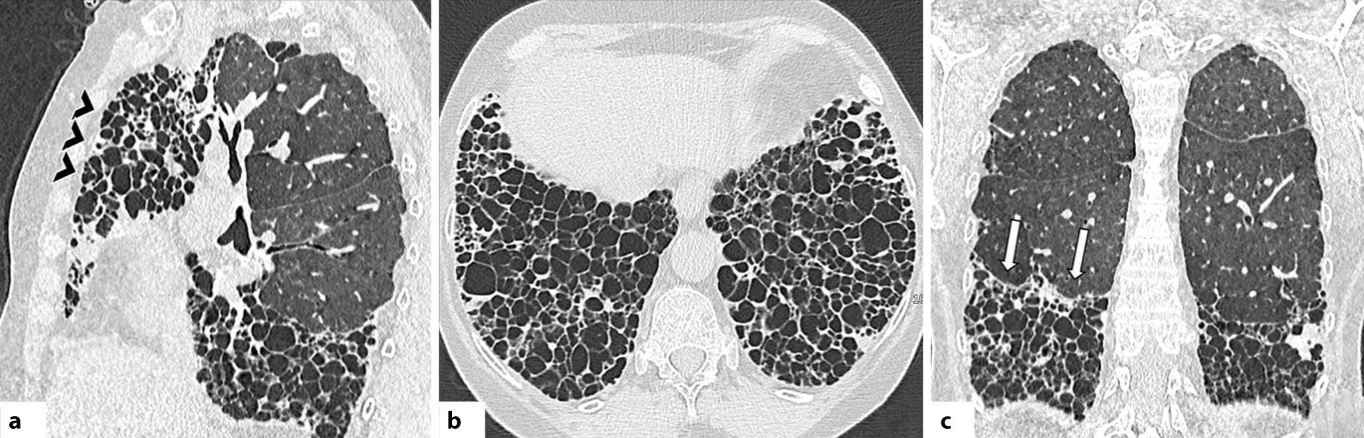
Hochauflösende Computertomographie (HRCT) von Patienten mit rheumatoider Arthritis und interstitieller Lungenerkrankung (ILD; Muster einer gewöhnlichen interstitiellen Pneumonie [UIP]), die drei für CTD-UIP typische Zeichen zeigen. **a** Sagittale Rekonstruktion: „anterior upper lobe sign“ (*schwarze Pfeilköpfe*) – Fibrose betont in den vorderen Anteilen der Oberlappen. **b** Axiale Rekonstruktion: „exuberant honeycombing“ – vergrößerte Zysten mit ausgedehntem Honeycombing. **c** Koronale Rekonstruktion: „straight edge sign“ (*weiße Pfeile*) – Fibrose überwiegend basal betont mit scharfer horizontaler Abgrenzung zum normalen Lungengewebe

Obwohl sich CTD-UIP und IPF-UIP radiologisch nicht sicher unterscheiden lassen, beschreiben Chung et al. 3 CT-Zeichen, die bevorzugt bei CTD-UIP auftreten:

- „anterior upper lobe sign“,
- „exuberant honeycombing“,
- „straight edge sign“ (Abb. 3; [39]).

Das „anterior upper lobe sign“ beschreibt ein Verteilungsmuster der Fibrose, bei dem sich fibrotische Veränderungen nicht nur in den basalen und dorsalen Lungenabschnitten finden, sondern auch in den ventralen Anteilen der Oberlappen – bei gleichzeitiger relativer Aussparung der übrigen Oberlappenareale [39]. Das „exuberant honeycombing“ ist gekennzeichnet durch eine ausgeprägtes Honeycombing mit vergrößerten Zysten (> 10 mm) und ausgedehnter Verteilung (> 70 % des fibrotischen Lungenanteils; [1, 39]). Das „straight edge sign“ zeigt eine basal betonte, horizontal scharf begrenzte Fibrose, die sich in der koronaren Rekonstruktion besonders gut erkennen lässt [1, 39].

Diese Zeichen könnten aus einer Progression von NSIP in UIP resultieren. So reflektiert das „exuberant honeycombing“ möglicherweise die ursprünglich homogene Verteilung der NSIP, die sich bei Verschlechterung flächenhaft in Honeycombing umwandelt [39]. Das „straight edge sign“ wird mit der peribronchovaskulären Verteilung der NSIP assoziiert. Im Krankheitsverlauf kann sich die Fibrose über die lateralen Begrenzungen der Lunge hinaus ausdehnen, was möglicherweise zum Auftreten dieses Zeichens führt [39]. Das Vorliegen eines UIP-Musters bei CTD hat prognostische Bedeutung. Bei RA ist UIP mit einer schlechteren Prognose assoziiert als andere ILD-Muster – auch wenn die Gesamtprognose besser bleibt als bei der IPF-bedingten UIP [40].

#### Organisierende Pneumonie

Die organisierende Pneumonie ist ein klinisch, radiologisch und histologisch definiertes Muster, das eine unspezifische Reaktion des Lungenparenchyms auf unterschiedliche Schädigungsursachen widerspiegelt [41]. Sie kann idiopathisch (kryptogene OP; [32]) oder sekundär bei Infektionen, Medikamententoxizität oder im Rahmen von CTD auftreten [41].

**Abb. 4:**
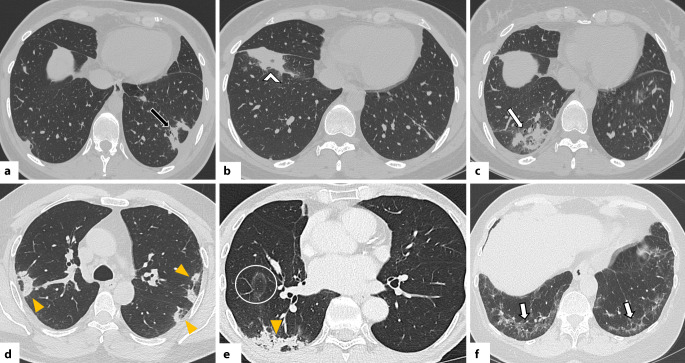
Axiale Aufnahmen der hochauflösenden Computertomographie (CT) zeigen für eine organisierende Pneumonie (OP) typische Befunde. **a** Periphere Konsolidierung im linken Unterlappen mit perilobulärem Muster (*schwarzer Pfeil*), vereinbar mit einer OP. **b**–**c** Verlaufskontrollen demonstrieren die typische Entwicklung der OP: migratorisches Verhalten und Wiederauftreten in anderen Arealen (*weißer Pfeilkopf*, *weißer Pfeil*). **d**, **e** Periphere Konsolidierungen mit perilobulärem Muster (*gelbe Pfeilköpfe*) sowie ein „reverse-halo sign“ (sog. „Atoll sign“, *weißer Kreis*). **f** OP-Muster mit Fibrose bei einem Patienten mit einem Antisynthetase-Syndrom: beidseitige lineare Verdichtungen in perilobulärer Verteilung (*weiße Pfeile*)

**Abb. 5:**
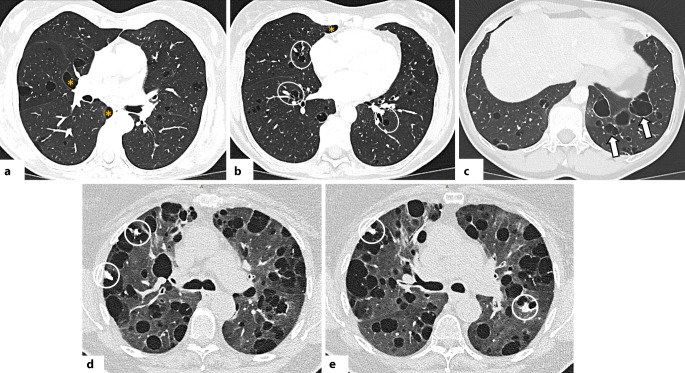
**a**–**c** Axiale Aufnahmen der hochauflösenden Computertomographie (HRCT): Muster einer lymphozytischen interstitiellen Pneumonie (LIP) bei einer Patientin mit Sjögren-Syndrom. Die Zysten sind dünnwandig und multiseptiert (*weiße Pfeile*), in zufälliger Verteilung und vorwiegend entlang peribronchovaskulärer Bündel (*weiße Kreise*) sowie subpleural bis hin zu mediastinalen Oberflächen lokalisiert (*gelbe Sternchen*). **d**, **e** Axiale HRCT-Aufnahmen bei einer Patientin mit Sjögren-Syndrom zeigen multiple verkalkte Noduli (*weiße Kreise*) in Kombination mit dünnwandigen Zysten, diese Befunde sprechen für eine pulmonale Amyloidose im Rahmen einer zugrundeliegenden LIP

In der HRCT zeigt sich die OP in einem breiten morphologischen Spektrum. Am häufigsten treten periphere und/oder peribronchovaskuläre Konsolidierungen auf, meist bilateral, asymmetrisch verteilt und mit Betonung der Unterlappen [42]. Diese Veränderungen sind häufig mit Milchglasverdichtungen kombiniert; innerhalb der Konsolidierungen können Luftbronchogramme erkennbar sein. Typisch ist auch ein migratorisches Verhalten der Veränderungen mit Rückbildung unter Steroidtherapie und Neuauftreten an anderen Stellen (Abb. 4; [42, 43]). Ein weiteres typisches Merkmal ist das perilobuläre Muster mit unscharfen, bogenförmigen („arcade-like“) Verdichtungen, meist subpleural gelegen und von belüftetem Lungengewebe umgeben [42, 43]. Das „reverse halo sign“ (auch „Atoll sign“) beschreibt ein Areal mit Milchglasverdichtungen, das ringförmig (komplett oder unvollständig) von einer Konsolidierung umgeben ist [27]. Obwohl nicht pathognomonisch, ist dieses Zeichen ein wichtiger, diagnostischer Hinweis auf eine OP (Abb. 5; [42, 43]). Seltenere Muster umfassen noduläre Veränderungen, lineare und bandförmige Verdichtungen, fokale Läsionen, mikronoduläre Veränderungen, das sog. Crazy-paving-Muster und eine fortschreitende Fibrose [42, 43]. Es ist hervorzuheben, dass die OP mit anderen Mustern interstitieller Pneumonien, insbesondere der NSIP, überlappen kann. In manchen Fällen bildet sich die OP nicht vollständig zurück und geht in ein fibrotisches Muster über [43].

#### Lymphozytäre interstitielle Pneumonie

Die lymphozytäre interstitielle Pneumonie ist durch eine inflammatorische Reaktion des bronchusassoziierten lymphatischen Gewebes (BALT) gekennzeichnet, die in einer diffusen polyklonalen interstitiellen Infiltration resultiert [44]. Sie stellt das fortgeschrittene Stadium eines pathophysiologischen Kontinuums dar, das von der follikulären Bronchiolitis (FB) – ausgelöst durch BALT-Hyperplasie – bis zur interstitiellen Beteiligung reicht [45]. Die LIP tritt selten idiopathisch auf; häufiger tritt sie im Zusammenhang mit CTD oder Immundefekten wie dem humanen Immundefizienz-Virus (HIV) in Erscheinung [44]. Im Rahmen der CTD wird die LIP am häufigsten beim Sjögren-Syndrom beobachtet, seltener auch bei der RA oder dem systemischem Lupus erythematodes (SLE; [45]).

In der HRCT zeigt die LIP typischerweise Milchglasverdichtungen und zentrilobuläre milchglasartige Noduli verschiedener Größe mit bevorzugter Verteilung entlang des perilymphatischen Interstitiums (d. h. bronchovaskuläre Bündel, interlobuläre Septen, subpleurale Regionen; [1, 46]). Die pulmonalen Noduli werden als Ausdruck einer lymphozytären Bronchiolitis gedeutet; ihre peribronchovaskuläre Lokalisation kann zu inhomogener Lungendichte infolge von Air-Trapping führen [1, 46].

**Abb. 6:**
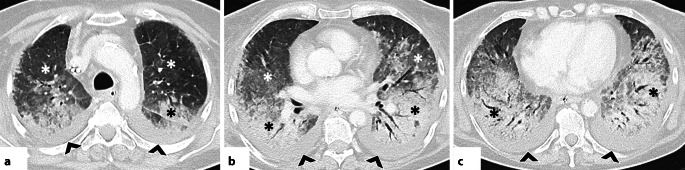
**a**–**c** Axiale Aufnahmen der hochauflösenden Computertomographie (HRCT): diffuse Milchglasverdichtungen (*weiße Sternchen*) in Kombination mit ausgedehnten Konsolidierungen (*schwarze Sternchen*), vorwiegend in den Unterlappen und abhängigen Lungenabschnitten. Zudem ist beidseitig ein Pleuraerguss erkennbar (*schwarze Pfeilköpfe*). Die Befunde entsprechen einem diffusen alveolären Schaden (DAD) im Rahmen einer vorbestehenden systemischen Sklerose

Ein typischer Befund bei der LIP sind dünnwandige Zysten (meist ≤30 mm), häufig mehrfach septiert, zufällig verteilt und oft von exzentrisch verlaufenden Gefäßen begleitet. Sie zeigen sich bevorzugt entlang der bronchovaskulären Bündel oder subpleural im mediastinalen Bereich (Abb. 6; [45]). Weitere HRCT-Zeichen umfassen eine Verdickung bronchovaskulärer Bündel und interlobulärer Septen, subpleurale Noduli und eine Vergrößerung mediastinaler Lymphknoten [1, 44, 46].

Die LIP kann ferner mit einer nodulären Amyloidose assoziiert sein, die sich als multiple, unterschiedlich große Noduli mit punktförmigen oder zentralen Verkalkungen nahe den Zysten manifestiert [46].

Wichtig ist, dass das LIP-Muster mit einer malignen Transformation – insbesondere einem MALT-Lymphom („mucosa-associated lymphoid tissue lymphoma“) – assoziiert sein kann. Das Auftreten größerer Noduli, Konsolidierungen oder einer Pleurabeteiligung sollte daher an eine lymphomatöse Entartung denken lassen [1, 44, 46].

#### Diffuser Alveolarschaden

Der diffuse Alveolarschaden ist das häufigste histologische Muster bei einem akuten Atemnotsyndrom (ARDS; [47]). Sie kann durch zahlreiche Ursachen ausgelöst werden – z. B. Medikamente, Sepsis, Trauma – oder idiopathisch auftreten (akute interstitielle Pneumonie, AIP; [47, 48]). Auch bei CTD, insbesondere bei IIMs, wurde über akute respiratorische Verschlechterungen durch DAD berichtet [47]. In der Studie von Parambil et al. zeigte sich der DAD sowohl als eine akute Exazerbation einer vorbestehenden CTD-ILD (ähnlich wie bei IPF) als auch als De-novo-Manifestation im Rahmen einer CTD – jeweils mit ungünstiger Prognose [47].

**Abb. 7:**
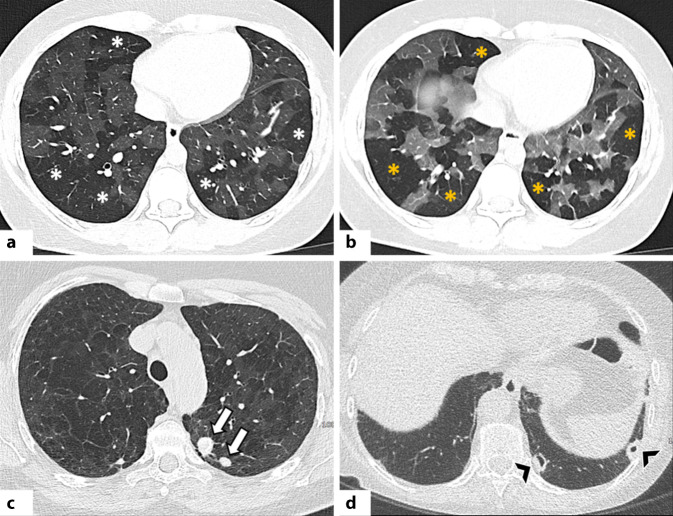
Obliterierende Bronchiolitis bei einer Patientin mit rheumatoider Arthritis (RA). **a** Axiale hochauflösende Computertomographie (HRCT) in Inspiration zeigt eine Mosaikperfusion bzw. -muster mit Arealen verminderter Dichte (*weiße Sternchen*) zwischen normal belüftetem Lungenparenchym. **b** In der Exspirationsaufnahme bestätigen sich diese Befunde als Areale von Air-Trapping (*gelbe Sternchen*). Rheumatoide Knötchen sind in **c** und **d** deutlich erkennbar. In **c** zeigen sich runde, periphere, solide Knötchen (*weiße Pfeile*) im apikoposterioren Segment des linken Oberlappens, während Bild **d** subpleurale, kavernierte Knötchen (*schwarze Pfeilspitzen*) im linken kostodiaphragmalen Winkel zeigt

Die HRCT-Merkmale eines CTD-assoziierten DAD unterscheiden sich nicht von jenen anderer Genese und variieren je nach Krankheitsstadium [47]. In der Frühphase finden sich typischerweise fleckige, bilaterale Milchglasverdichtungen in geografischer Verteilung [1, 48]. Im Verlauf gehen diese in ausgedehnte Konsolidierungen über, die bevorzugt die Unterlappen und gravitationsabhängigen Lungenabschnitte betreffen (Abb. 7; [1, 48]). In der fibrotischen Spätphase zeigen sich Zeichen des Parenchymumbaus wie Retikulationen, Traktionsbronchiektasen und Volumenverlust, meist in nicht gravitationsabhängigen Arealen [1, 48].

## Wichtige Bindegewebserkrankungen und ihr Muster der Lungenmanifestation

### Rheumatoide Arthritis

Die Lungenbeteiligung stellt eine wesentliche Todesursache bei der rheumatoiden Arthritis dar. Die ILD ist die häufigste pulmonale Manifestation der RA, mit einer Prävalenz zwischen 10 und 30 %, abhängig von den zugrundeliegenden Diagnosekriterien [1, 9]. Risikofaktoren für die Entwicklung einer RA-ILD sind u. a. Rauchen, höheres Alter, männliches Geschlecht und erhöhte Entzündungsparameter [49]. Die HRCT ist die sensitivste Methode zur Erkennung einer RA-ILD [1]. Am häufigsten zeigt sich das UIP-Muster, gefolgt von dem NSIP-Muster. Weniger häufig sind die OP, die LIP und der DAD [30, 31, 49]. Typische CT-Zeichen einer RA-UIP sind das „anterior upper lobe sign“, das „exuberant honeycombing“ und das „straight edge sign“ [39].

Der DAD kann entweder als Erstmanifestation einer RA-ILD auftreten oder, häufiger, als akute Exazerbation einer vorbestehenden fibrotischen ILD, mit einer Exazerbationsrate ähnlich der von IPF [31]. Radiologisch ist der DAD bzw. die Exazerbation meist nicht von einer Pneumonie zu unterscheiden [31]. Weitere mögliche Befunde im Rahmen einer ILD sind Atemwegserkrankungen wie die obliterative Bronchiolitis (OB) und die follikuläre Bronchiolitis (FB) sowie rheumatoide Knoten [1, 31].

**Abb. 8:**
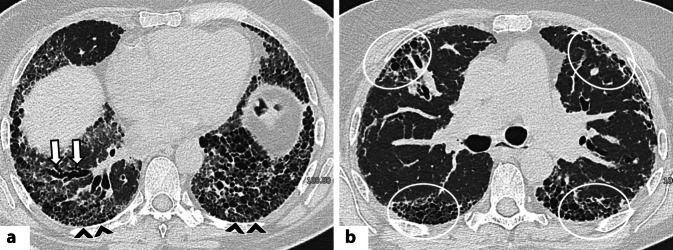
**a** Axiale hochauflösende Computertomographie (HRCT) einer Patientin mit systemischer Sklerose (SSc): Übergang von einem NSIP-Muster (nichtspezifische interstitielle Pneumonie) – erkennbar an zentralen Bronchiektasen im rechten Unterlappen (*weiße Pfeile*) umgeben von Milchglasverdichtungen – hin zu einem UIP-Muster (gewöhnliche interstitielle Pneumonie) mit bilateralem, subpleuralem Honeycombing (*schwarze Pfeilköpfe*). **b** Axiale HRCT-Aufnahme bei einer Patientin mit SSc mit dem sog. „four-corner sign“: Zeichen einer pulmonalen Fibrose (einschließlich Honeycombing) in den vier „Ecken“ der Lunge – anterolaterale Areale der Oberlappen und posterosuperioren Arealen der Unterlappen (*weiße Kreise*)

Die OB ist durch eine Fibrose der Bronchialwände der kleinen Atemwege charakterisiert und ist gekennzeichnet durch ein Mosaikmuster in der Inspirationsphase sowie Air-Trapping in der Exspirationsphase (Abb. 8a, b; [1, 31]). Weitere Zeichen können eine Wandverdickung der Bronchien, eine proximale Dilatation der Atemwege sowie freistehende Bronchiektasen sein. Die FB entsteht durch lymphatische Hyperplasie in den Bronchiolen und zeigt sich in der HRCT typischerweise als zentrilobuläre Noduli [1, 31].

Rheumaknoten in der Lunge erscheinen in der CT als multiple, runde oder lobulierte Läsionen variabler Größe, meist peripher gelegen und oft kavitiert (Abb. 8c, d; [49]). Interessanterweise zeigte eine große Studie von Jacob et al., dass 27 % der RA-ILD-Patienten Emphyseme aufwiesen, ohne Rauchvorgeschichte [50].

### Systemische Sklerose

Die systemische Sklerose wird anhand des Ausmaßes der Hautbeteiligung in limitierte und diffuse kutane Formen unterteilt [31]. Pulmonale Manifestationen sind häufig, mit einer Prävalenz zwischen über 22 und bis zu 80 % [1, 51]. Hauptmanifestationen sind die ILD und die pulmonale Hypertonie (PH; [1, 51]). Die ILD tritt bei SSc häufiger auf als bei anderen CTD, besonders bei der diffusen Form [31]. Weitere Risikofaktoren sind männliches Geschlecht und das Vorhandensein von Anti-Scl-70-Antikörpern [52].

**Abb. 9:**
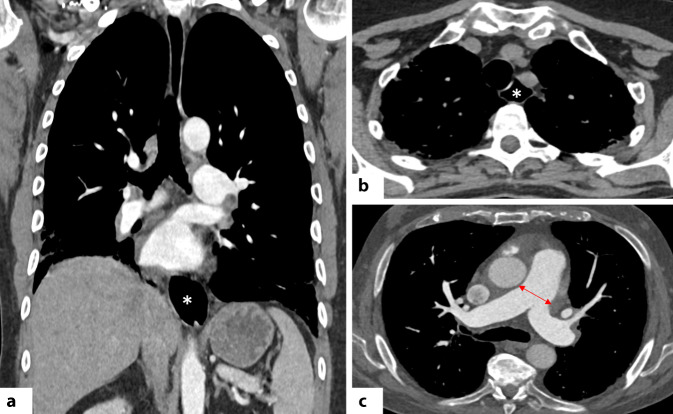
Koronale und axiale CT-Aufnahmen bei Patientinnen mit systemischer Sklerose zeigen eine Ösophagusdilatation (*weiße Sternchen*) im distalen (**a**) bzw. oberen Mediastinum (**b**). Axiale kontrastverstärkte CT (**c**) bei einer Patientin mit SSc mit pulmonaler Hypertonie (PH): deutliche Erweiterung des Truncus pulmonalis (*roter Pfeil*). Ein Trunkusdurchmesser >29 mm sowie ein Verhältnis Durchmesser zu Pulmonalarterie/aufsteigender Aorta (PA/A) > 1 gelten als hinweisend auf eine PH

Das häufigste HRCT-Muster bei der SSc ist die NSIP (etwa 75–80 %), gefolgt von der UIP (10–15 %; [31, 52]). Das Vorliegen von Honeycombing kann auf eine fortgeschrittene Erkrankung hinweisen (Abb. 9a; [13]). Das „straight edge sign“ tritt bei der SSc häufiger auf als bei der RA [1].

**Abb. 10:**
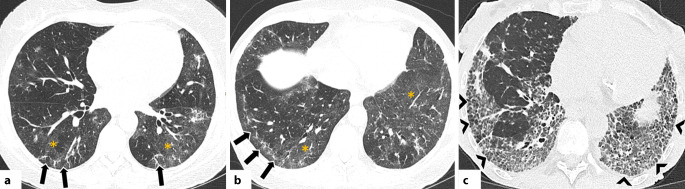
**a**, **b** Axiale hochauflösende Computertomographie (HRCT) zeigen bei einer Patientin mit einem Antisynthetase-Syndrom (Anti-Jo-1-Antikörper) diffuse, zentral betonte Milchglasverschattungen (*gelbe Sternchen*) sowie periphere lineare Verdichtungen in den dorsalen Abschnitten der Oberlappen und des rechten Unterlappens (*schwarze Pfeile*), hinweisend auf ein perilobuläres Verteilungsmuster. Diese Befunde sind vereinbar mit einem kombinierten NSIP-/OP-Muster (nichtspezifische und organisierende Pneumonie). **c** Zehn Jahre später zeigt die Verlaufskontrolle eine Progredienz zu einem UIP-Muster (gewöhnliche interstitielle Pneumonie) mit Milchglasverdichtungen (hier Hinweis auf feine Fibrose), Traktionsbronchiektasen und bilateralem subpleuralem Honeycombing (*schwarze Pfeilköpfe*) in den Mittel- und Unterlappen; zudem deutlicher Volumenverlust beider Unterlappen

Ein weiteres diagnostisches Merkmal ist das „four corner sign“: retikuläre Veränderungen, Milchglasverdichtungen und/oder Honeycombing, fokal lokalisiert in den „vier Ecken“ der Lunge, bilateral anterolateral in den oberen Mittellappen sowie posterosuperior in den Unterlappen (Abb. 9b; [1, 51]). Laut Walkoff et al. ist dieses Zeichen hochspezifisch (100 %) für eine SSc-ILD, jedoch wenig sensitiv (16 %; [51]. Andere Muster wie OP, DAD oder FB sind im Rahmen der SSc selten [1]. Weitere typische CT-Befunde sind eine ösophageale Dilatation (Abb. 10a, b) und eine Lymphadenopathie [31, 52]. Die ILD ist die führende Morbiditäts- und Mortalitätsursache bei der SSc, wobei kein signifikanter Unterschied in der Prognose zwischen UIP- und NSIP-Mustern festgestellt wurde [53].

Die PH hingegen wird häufiger bei der limitierten SSc beobachtet, isoliert oder kombiniert mit Fibrose [1]. Vier Formen der PH sind beschrieben: pulmonal-arterielle Hypertonie, chronisch-thromboembolische Erkrankung, sekundär bei ILD und sekundär bei der linksventrikulären Dysfunktion [31]. Auch wenn die Rechtsherzkatheterisierung der Goldstandard bleibt, können CT-Zeichen wie eine Vergrößerung der zentralen und proximalen Pulmonalarterien, Perikarderguss/-verdickung sowie ein Mosaikmuster Hinweise auf eine PH liefern (Abb. 10c; [1, 31]). Letzteres zeigt Areale verminderter Dichte mit verengten Gefäßen (reduzierte Perfusion) und dichtere Abschnitte mit Hyperämie [31]. Die Abgrenzung zur obliterativen Atemwegserkrankung erfolgt durch das Fehlen von Air-Trapping in der Exspirationsphase.

### Idiopathische inflammatorische Myopathien

Idiopathische inflammatorische Myopathien sind seltene Autoimmunerkrankungen, die durch eine Entzündung der Skelettmuskulatur mit konsekutiver Schwäche und Atrophie charakterisiert sind [54]. Zu den IIM zählen verschiedene Subtypen wie die Dermatomyositis (DM), das Antisynthetase-Syndrom (ASyS), die immunvermittelte nekrotisierende Myopathie (IMNM), die Einschlusskörpermyositis (IBM), die Overlap-Myositis (OM) und die Polymyositis (PM) [54, 55]. Die IIM gehen häufig mit systemischen Manifestationen einher, wobei die pulmonale Beteiligung zu den häufigsten und schwerwiegendsten zählt [54].

Zu den Lungenmanifestationen zählen Hypoventilation und respiratorisches Versagen durch muskuläre Beteiligung, ILD sowie Aspirationspneumonien infolge pharyngealer Muskelschwäche [1, 56]. Die Prävalenz der ILD bei IIM liegt zwischen 20 und 78 % und kann dem Beginn der Myositis vorausgehen [57]. Unter den Subtypen ist das ASyS am stärksten mit einer ILD assoziiert [58]. Die hypomyopathische DM, eine seltene Form der IIM mit Autoantikörpern gegen das Melanom-Differenzierungs-assoziierte Gen 5 (MDA-5), geht typischerweise mit einer rasch progredienten ILD einher [31]. Weitere Risikofaktoren für die Entwicklung einer ILD sind höheres Alter, Arthralgien/Arthritis, Raynaud-Phänomen sowie erhöhte Entzündungsparameter (BSG, CRP; [59]).

Die häufigsten HRCT-Muster bei den IIM sind die NSIP und die OP, meist in Kombination als NSIP/OP-Overlap [1, 30, 31]. NSIP und NSIP/OP werden häufig beim ASyS beobachtet, während bei Anti-MDA-5-positiven Patienten typischerweise die OP und Konsolidierungen auftreten [60]. Die OP bei den IIM zeigt häufig ein eher alveoläres und weniger bronchozentrisches Verteilungsmuster der Konsolidierungen im Vergleich zu der OP bei Nicht-IIM [61]. Ein UIP-Muster kann gelegentlich vorkommen, insbesondere bei Anti-Jo1-Antikörper-positiven Patienten [1, 60]. Der DAD wird nur selten beobachtet, verläuft dann aber meist rasch progredient [61].

### Systemischer Lupus erythematodes

Die pulmonale Beteiligung beim systemischen Lupus erythematodes betrifft am häufigsten die Pleura [12, 30, 31, 56]. Lungenmanifestationen treten bei 20–90 % der Patienten auf und können akut oder chronisch verlaufen [56]. Zu den akuten Formen zählen die pulmonale Hämorrhagie, die akute Lupus-Pneumonitis und das Shrinking-Lung-Syndrom, während chronische Verläufe meist durch eine ILD geprägt sind [1, 56]. Eine ILD tritt im Vergleich zu anderen CTD seltener auf, mit einer Prävalenz zwischen 0,5 und 17,5 % [62]. Risikofaktoren sind männliches Geschlecht, höheres Alter bei Krankheitsbeginn und eine Vorgeschichte einer Lupus-Pneumonitis [31]. Die NSIP ist das häufigste HRCT-Muster, gelegentlich treten auch eine OP, eine LIP und eine UIP auf [1, 63]. In der Kohorte von Brady et al. wurde eine fibrotische NSIP bei 38 % der Patienten beobachtet [62]. Die für CTD typischen fibrotischen Varianten wie das „anterior upper lobe sign“, das „straight edge sign“ und das „exuberant honeycombing“ wurden auch bei SLE beschrieben [63]. Zudem fanden Brady et al. zwei weitere charakteristische Bildmerkmale bei der SLE-ILD: eine heterogene Lungendestruktion (48 %) und eine inselartige Fibrose (62 %; [62]). Die heterogene Lungendestruktion bezeichnet geografische Areale von Narbenemphysem und Architekturstörung mit irregulären zystischen Veränderungen, die sich von Honeycombing abgrenzen lassen [62]. Die inselartige Fibrose beschreibt keilförmige, scharf begrenzte Areale zentraler Fibrose, meist in den oberen Segmenten der Unterlappen [62].

HRCT-Zeichen einer Lungenblutung sind bilaterale, zentrale, diffuse oder fleckige Milchglasverdichtungen, teils mit Konsolidierungen [63]. Bei passender Klinik (z. B. Hämoptysen, Hämoglobinfall) sollte eine Alveolarblutung erwogen werden [1, 63]. Die akute Lupus-Pneumonitis ist selten, aber potenziell letal, mit einer Häufigkeit von ca. 10 % [1, 63]. Die CT zeigt meist fleckige Milchglasverdichtungen und basale Konsolidierungen bei erhaltener Architektur [63]. Diese Befunde spiegeln eine alveoläre Entzündung, ein interstitielles Ödem und einen alveolären Kollaps wider [63]. Das Shrinking-Lung-Syndrom äußert sich durch progredienten Volumenverlust der Lunge ohne ersichtliche Ursache, oft mit einem Zwerchfellhochstand und einer basalen Atelektase in der CT [31]. Die Pathogenese ist ungeklärt; diskutiert werden eine respiratorische Myopathie, eine Phrenikusneuropathie, ein Surfactant-Mangel und pleurale Adhäsionen [64]. Sowohl die akute Lupus-Pneumonitis als auch das Shrinking-Lung-Syndrom sind Ausschlussdiagnosen [63]. Zudem sind SLE-Patienten aufgrund der immunologischen Dysregulation und immunsuppressiver Therapie anfälliger für opportunistische Infektionen [31]. Weitere pulmonale Komplikationen umfassen eine PH und Lungenembolien, meist im Zusammenhang mit Antiphospholipidantikörpern [1].

### Sjögren-Syndrom

Die berichtete Prävalenz der pulmonalen Beteiligung beim Sjögren-Syndrom variiert stark und liegt zwischen 9 und 75 % [65]. Lungenmanifestationen umfassen Atemwegserkrankungen (v. a. FB), ILD und lymphoproliferative Erkrankungen, die häufig in Kombination auftreten [1, 31]. Interstitielle Pneumonien sind relativ häufig und treten in ca. 25–50 % der Fälle auf [1, 9]. Eine ILD im Rahmen von SjD ist mit einer verkürzten Lebenserwartung assoziiert. Risikofaktoren für eine ILD-Entwicklung sind männliches Geschlecht, eine lange Krankheitsdauer, systemische Manifestationen und Anti-Ro52-(SSA-)Antikörper [65].

Die häufigsten HRCT-Muster bei SjD sind die NSIP und die LIP; seltener treten die OP und die UIP auf [1, 46]. Obwohl die LIP früher als häufigster Subtyp galt, zeigen neuere Studien, dass die NSIP vorherrschend ist [1, 66, 67]. Ein HRCT-Muster, das auf eine NSIP hinweist, korreliert eng mit der Histologie, sodass eine chirurgische Biopsie oft nicht erforderlich ist [1, 66]. Die HRCT kann auch helfen, eine SjD-ILD von anderen Ursachen des Sicca-Syndroms, insbesondere Sarkoidose, abzugrenzen [31].

Eine weitere häufige pulmonale Manifestation ist die noduläre Amyloidose mit multiplen, oft verkalkten Rundherden [31]. Diese Läsionen treten typischerweise vor dem Hintergrund einer LIP oder gemeinsam mit dünnwandigen Zysten auf [46].

Primäre pulmonale Lymphome können ebenfalls bei einem SjD auftreten, meist als Marginalzonenlymphome, welche vom MALT-Typ in den Atemwegen abstammen [31]. Typische HRCT-Befunde sind Konsolidierungen oder tumorartige Verdichtungen mit Luftbronchogrammen [31].

### Mischkollagenose

Der Begriff Mischkollagenose („mixed connective tissue disease“, MCTD), vormals auch als Overlap-Syndrom bezeichnet [68], beschreibt ein Krankheitsbild mit klinischen Überlappungen von SLE, SjD, RA und IIM [1, 2]. Patienten mit einer MCTD erfüllen nicht alle Klassifikationskriterien der anderen CTD [1]. Im Gegensatz zur undifferenzierten CTD zeichnet sich die MCTD durch das Vorhandensein von Anti-U1RNP-Antikörpern aus [1]. Pulmonale Manifestationen sind häufig, wobei eine ILD bei bis zu einem Drittel der Patienten auftritt [31, 69]. Zu den Risikofaktoren für die Entwicklung einer ILD bei MCTD gehören höheres Alter und Anti-Ro52-Antikörper [1, 68].

Die HRCT-Befunde bei MCTD-ILD sind heterogen und ähneln häufig jenen bei SLE, SSc und PM [1, 30]. Das häufigste radiologische Muster ist die NSIP (v. a. die fibrotische Form), gefolgt von der OP und der UIP [31, 69]. Auch das DAD-Muster wurde beschrieben und könnte akute Exazerbationen widerspiegeln [1, 31]. Wie bei anderen CTD ist auch bei MCTD-ILD die ILD mit einer erhöhten Mortalität assoziiert [69].

## Fazit für die Praxis

- Die Lungenbeteiligung ist eine häufige Manifestation von Bindegewebserkrankungen und stellt eine der Hauptursachen für Morbidität und Mortalität dar.
- Die hochauflösende Computertomographie (HRCT) spielt eine zentrale Rolle in der Diagnostik, im Therapiemonitoring und in der Verlaufskontrolle von Kollagenosen mit interstitiellen Lungenerkrankungen (CTD-ILD).
- Aufgrund der komplexen und heterogenen Präsentation dieser Krankheitsgruppe, die sich überlappen, weiterentwickeln oder akut manifestieren kann, ist ein multidisziplinärer Ansatz entscheidend für die Festlegung der endgültigen Diagnose.
